# Tetra­kis(1*H*-benzimidazole-κ*N* ^3^)(nitrato-κ*O*)copper(II) nitrate

**DOI:** 10.1107/S1600536811018885

**Published:** 2011-05-25

**Authors:** Fu-Lin Zhou, Seik Weng Ng

**Affiliations:** a Department of Applied Chemistry, Yuncheng University, Yuncheng, Shanxi 044000, People’s Republic of China; b Department of Chemistry, University of Malaya, 50603 Kuala Lumpur, Malaysia

## Abstract

In the title salt, [Cu(NO_3_)(C_7_H_6_N_2_)_4_]NO_3_, one nitrate anion is coordinated to the Cu^II^ atom, which is also coordinated by the N atoms of four *N*-heterocycles. The geometry at the metal atom is a square pyramid in which the O atom of the anion occupies the apical position [Cu—O = 2.468 (5) and 2.590 (7) Å in the two independent formula units]. The cation lies on a twofold rotation axis; the coordinated nitrate anion is also disordered about this symmetry element. The lattice anion is also disordered about a twofold rotation axis. In the crystal, the cations are linked to the coordinated and free anions by N—H⋯O hydrogen bonds.

## Related literature

For selected Cu^II^ adducts of imidazole and benzimidazole, see: Dobrzyńska *et al.* (2010 ▶); McFadden *et al.* (1975 ▶, 1976 ▶); Sieroń (2007 ▶).
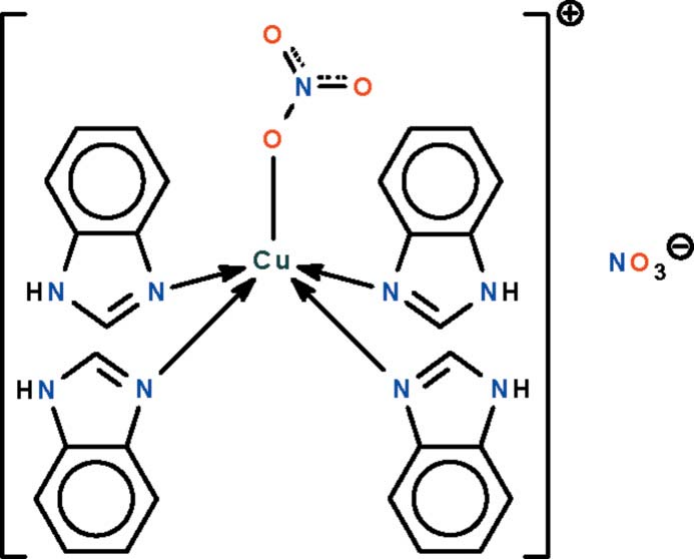


## Experimental

### 

#### Crystal data

[Cu(NO_3_)(C_7_H_6_N_2_)_4_]NO_3_
*M* *_r_* = 660.12
Orthorhombic, 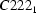

*a* = 15.7181 (2) Å
*b* = 24.9338 (3) Å
*c* = 15.1048 (2) Å
*V* = 5919.75 (13) Å^3^
*Z* = 8
Cu *K*α radiation
μ = 1.56 mm^−1^
*T* = 293 K
0.10 × 0.08 × 0.06 mm

#### Data collection

Agilent Xcalibur Eos Gemini diffractometer
Absorption correction: multi-scan (*CrysAlis PRO*; Agilent, 2010 ▶) *T*_min_ = 0.860, *T*_max_ = 0.912
7285 measured reflections
5038 independent reflections
4779 reflections with *I* > 2σ(*I*)
*R*_int_ = 0.011

#### Refinement

*R*[*F* ^2^ > 2σ(*F* ^2^)] = 0.040
*wR*(*F* ^2^) = 0.117
*S* = 1.03
5038 reflections
431 parameters
130 restraints
H-atom parameters constrained
Δρ_max_ = 0.32 e Å^−3^
Δρ_min_ = −0.31 e Å^−3^
Absolute structure: Flack (1983 ▶), 1676 Friedel pairs
Flack parameter: −0.05 (3)

### 

Data collection: *CrysAlis PRO* (Agilent, 2010 ▶); cell refinement: *CrysAlis PRO*; data reduction: *CrysAlis PRO*; program(s) used to solve structure: *SHELXS97* (Sheldrick, 2008 ▶); program(s) used to refine structure: *SHELXL97* (Sheldrick, 2008 ▶); molecular graphics: *X-SEED* (Barbour, 2001 ▶); software used to prepare material for publication: *publCIF* (Westrip, 2010 ▶).

## Supplementary Material

Crystal structure: contains datablocks global, I. DOI: 10.1107/S1600536811018885/zs2111sup1.cif

Structure factors: contains datablocks I. DOI: 10.1107/S1600536811018885/zs2111Isup2.hkl

Additional supplementary materials:  crystallographic information; 3D view; checkCIF report

## Figures and Tables

**Table 1:** Hydrogen-bond geometry (Å, °)

*D*—H⋯*A*	*D*—H	H⋯*A*	*D*⋯*A*	*D*—H⋯*A*
N2—H2⋯O1^i^	0.88	2.21	3.069 (9)	167
N2—H2⋯O3^ii^	0.88	1.89	2.742 (9)	164
N4—H4⋯O5^ii^	0.88	2.24	2.964 (9)	139
N4—H4⋯O6^iii^	0.88	2.10	2.955 (7)	163
N6—H6⋯O7	0.88	2.23	2.998 (14)	146
N8—H8⋯O10	0.88	1.91	2.79 (2)	173
N8—H8⋯O12^iv^	0.88	1.87	2.75 (3)	175

Symmetry codes: (i) 
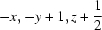
; (ii) 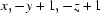; (iii) 
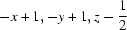
; (iv) 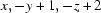.

**Fig. 1.**
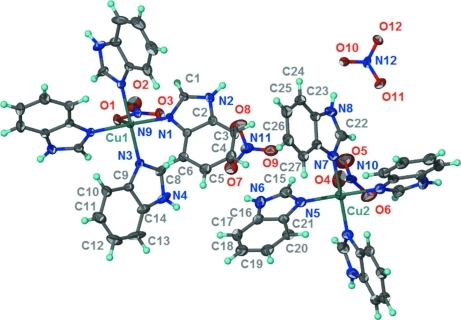
Thermal ellipsoid plot (Barbour, 2001) of two independent formula units of [Cu(NO3)(C7H6N2)4] NO3 at the 30% probability level with hydrogen atoms drawn as spheres of arbitrary radius. Symmetry-related benzimidazole ligands are not labeled.

## supplementary crystallographic information

### Comment

Whereas the coordination sphere of the copper(II) ion (and probably most first-row transition metal ions) readily accomodates four to six imidazole ligands, the space sphere is not large enough to fit a similar number of the related benzimidazole ligands. In the copper nitrate–tetrakis(imidazole) adduct, the nitrate ion is involved in coordination (McFadden *et al.*, 1976) but in the hexakis(imidazole) adduct, the nitrate ion is not (McFadden *et al.*, 1975). The tetrakis(benzimidazole)copper species has been reported for the perchlorate (Dobrzyńska *et al.*, 2010) and the sulfate (Sierón, 2007) only. These two, as well the present nitrate (Scheme I), have the Cu^II^ atom in a square pyramidal geometry but with the square pyramid being distorted because the ligand crowds out the donor atom of the anion.

In the crystal of the salt, Cu(NO_3_)(C_7_H_6_N_2_)_4_^.^NO_3_, one nitrate anion is coordinated to the Cu^II^ atom, which is also coordinated by the N atoms of four *N*-heterocycles (Fig. 1). The geometry at the metal atom is a square pyramid in which the O atom of the anion occupies the apical position [Cu–O 2.468 (5), 2.590 (7) Å in the two independent formula units]. The coordinated and lattice nitrates are disordered about this symmetry element. The disorder, which requires space for the anions to rattle around, probably accounts for the somewhat large solvent-accessible voids. Nevertheless, the cations interact with the anions through N–H···O hydrogen bonds (Table 1).

### Experimental

Copper nitrate trihydrate (0.246 g), 1,3-benzimidazole (0.473 g) and water (2 ml) were placed in a 25-ml, Teflon-lined Parr bomb. The bomb was heated at 413 K for 5 days. Blue prismatic crystals were isolated after the bomb was cooled to room temperature over the course of a day.

### Refinement

Carbon-bound H-atoms were placed in calculated positions [C—H = 0.93 Å, *U*_iso_(H) = 1.2*U*_eq_(C)] and were included in the refinement in the riding model approximation. The aromatic rings were refined as rigid hexagons with 1.39 Å sides. The four nitrate ions were allowed to refine off twofold rotation axes subject to distance restraints of N–O = 1.24±0.01 Å and O···O 2.15 Å. The four-atom nitrate units were restrained to lie on a plane. The anisotropic displacement parameters of the nitrate ions were restrained to be nearly isotropic. The absolute structure parameter was refined from 1676 Friedel pairs.

### Crystal data

**Table:**

[Cu(NO_3_)(C_7_H_6_N_2_)_4_]NO_3_	*F*(000) = 2712
*M**_r_* = 660.12	*D*_x_ = 1.481 Mg m^−^^3^
Orthorhombic, *C*222_1_	Cu *K*α radiation, λ = 1.5418 Å
Hall symbol: C 2c 2	Cell parameters from 5605 reflections
*a* = 15.7181 (2) Å	θ = 2.9–70.5°
*b* = 24.9338 (3) Å	µ = 1.56 mm^−^^1^
*c* = 15.1048 (2) Å	*T* = 293 K
*V* = 5919.75 (13) Å^3^	Prism, blue
*Z* = 8	0.10 × 0.08 × 0.06 mm

### Data collection

**Table:**

Agilent Xcalibur Eos Gemini diffractometer	5038 independent reflections
Radiation source: Enhance (Cu) X-ray Source	4779 reflections with *I* > 2σ(*I*)
graphite	*R*_int_ = 0.011
Detector resolution: 16.0356 pixels mm^-1^	θ_max_ = 70.7°, θ_min_ = 3.3°
ω scans	*h* = −17→19
Absorption correction: multi-scan (*CrysAlis PRO*; Agilent, 2010)	*k* = −30→12
*T*_min_ = 0.860, *T*_max_ = 0.912	*l* = −18→18
7285 measured reflections	

### Refinement

**Table:**

Refinement on *F*^2^	Secondary atom site location: difference Fourier map
Least-squares matrix: full	Hydrogen site location: inferred from neighbouring sites
*R*[*F*^2^ > 2σ(*F*^2^)] = 0.040	H-atom parameters constrained
*wR*(*F*^2^) = 0.117	*w* = 1/[σ^2^(*F*_o_^2^) + (0.081*P*)^2^ + 2.0279*P*] where *P* = (*F*_o_^2^ + 2*F*_c_^2^)/3
*S* = 1.03	(Δ/σ)_max_ = 0.001
5038 reflections	Δρ_max_ = 0.32 e Å^−^^3^
431 parameters	Δρ_min_ = −0.31 e Å^−^^3^
130 restraints	Absolute structure: Flack (1983), 1676 Friedel pairs
Primary atom site location: structure-invariant direct methods	Flack parameter: −0.05 (3)

### Fractional atomic coordinates and isotropic or equivalent isotropic displacement parameters (Å^2^)

**Table:**

	*x*	*y*	*z*	*U*_iso_*/*U*_eq_	Occ. (<1)
Cu1	0.0000	0.417817 (18)	0.2500	0.03722 (14)	
Cu2	0.5000	0.38135 (2)	0.7500	0.05537 (17)	
O1	−0.0129 (5)	0.5148 (3)	0.1902 (6)	0.0616 (18)	0.50
O2	−0.0161 (7)	0.59502 (16)	0.2397 (10)	0.101 (3)	0.50
O3	0.0386 (5)	0.5315 (3)	0.3167 (6)	0.074 (2)	0.50
O4	0.4732 (5)	0.47882 (18)	0.7442 (8)	0.107 (2)	0.50
O5	0.4466 (6)	0.5603 (3)	0.7658 (10)	0.156 (4)	0.50
O6	0.5717 (4)	0.5318 (3)	0.7891 (6)	0.117 (3)	0.50
O7	0.2850 (7)	0.4949 (5)	0.4463 (6)	0.089 (3)	0.50
O8	0.1786 (3)	0.4969 (4)	0.5351 (5)	0.110 (3)	0.50
O9	0.3011 (5)	0.5214 (4)	0.5815 (5)	0.083 (2)	0.50
O10	0.1531 (11)	0.4740 (12)	0.9487 (14)	0.056 (3)	0.50
O11	0.2713 (2)	0.4965 (11)	1.0107 (14)	0.064 (3)	0.50
O12	0.1518 (13)	0.5215 (12)	1.0668 (14)	0.059 (4)	0.50
N1	0.03749 (15)	0.40674 (10)	0.37505 (15)	0.0423 (5)	
N2	0.04470 (17)	0.42208 (11)	0.51886 (16)	0.0509 (6)	
H2	0.0323	0.4352	0.5714	0.061*	
N3	0.12264 (13)	0.41851 (10)	0.21267 (16)	0.0436 (5)	
N4	0.25856 (17)	0.43865 (13)	0.2204 (3)	0.0744 (11)	
H4	0.3059	0.4544	0.2377	0.089*	
N5	0.46012 (19)	0.37857 (12)	0.6251 (2)	0.0603 (7)	
N6	0.4071 (2)	0.40852 (14)	0.5002 (2)	0.0744 (9)	
H6	0.3816	0.4302	0.4626	0.089*	
N7	0.37999 (19)	0.37880 (11)	0.7923 (2)	0.0598 (7)	
N8	0.2594 (2)	0.40803 (13)	0.8488 (2)	0.0708 (8)	
H8	0.2231	0.4290	0.8766	0.085*	
N9	0.0030 (5)	0.54803 (13)	0.2490 (8)	0.0462 (9)	0.50
N10	0.4980 (6)	0.52280 (17)	0.7649 (6)	0.059 (3)	0.50
N11	0.2553 (4)	0.5029 (6)	0.5215 (4)	0.066 (3)	0.50
N12	0.1927 (3)	0.5001 (13)	1.0062 (17)	0.048 (2)	0.50
C1	0.0063 (2)	0.43455 (11)	0.44316 (18)	0.0478 (6)	
H1	−0.0372	0.4597	0.4383	0.057*	
C2	0.10595 (12)	0.38578 (8)	0.50119 (11)	0.0494 (6)	
C3	0.16641 (15)	0.35976 (10)	0.55281 (9)	0.0660 (9)	
H3	0.1702	0.3672	0.6130	0.079*	
C4	0.22118 (14)	0.32267 (10)	0.51443 (14)	0.0723 (10)	
H4A	0.2616	0.3053	0.5490	0.087*	
C5	0.21549 (13)	0.31161 (9)	0.42443 (14)	0.0680 (9)	
H5	0.2521	0.2868	0.3988	0.082*	
C6	0.15502 (13)	0.33764 (8)	0.37281 (10)	0.0515 (7)	
H6A	0.1512	0.3302	0.3126	0.062*	
C7	0.10025 (11)	0.37472 (7)	0.41119 (11)	0.0428 (6)	
C8	0.18048 (17)	0.44833 (12)	0.2533 (3)	0.0563 (7)	
H8A	0.1687	0.4726	0.2983	0.068*	
C9	0.16669 (11)	0.38703 (9)	0.15231 (13)	0.0509 (7)	
C10	0.13867 (13)	0.34785 (10)	0.09369 (15)	0.0656 (9)	
H10	0.0813	0.3389	0.0914	0.079*	
C11	0.1964 (2)	0.32205 (10)	0.03854 (15)	0.0909 (16)	
H11	0.1777	0.2958	−0.0007	0.109*	
C12	0.28222 (19)	0.33543 (13)	0.04201 (19)	0.0989 (18)	
H12	0.3209	0.3182	0.0051	0.119*	
C13	0.31024 (11)	0.37460 (13)	0.1006 (2)	0.109 (2)	
H13	0.3676	0.3836	0.1029	0.131*	
C14	0.25248 (13)	0.40040 (10)	0.15578 (17)	0.0682 (10)	
C15	0.4189 (3)	0.41740 (16)	0.5867 (3)	0.0720 (10)	
H15	0.3998	0.4479	0.6161	0.086*	
C16	0.44165 (15)	0.36015 (8)	0.48107 (15)	0.0637 (8)	
C17	0.44827 (18)	0.32937 (11)	0.40466 (12)	0.0818 (12)	
H17	0.4260	0.3420	0.3516	0.098*	
C18	0.48821 (18)	0.27966 (11)	0.40755 (14)	0.0836 (12)	
H18	0.4926	0.2591	0.3564	0.100*	
C19	0.52152 (16)	0.26073 (8)	0.48686 (18)	0.0764 (11)	
H19	0.5482	0.2275	0.4888	0.092*	
C20	0.51490 (15)	0.29151 (8)	0.56328 (14)	0.0638 (9)	
H20	0.5372	0.2788	0.6163	0.077*	
C21	0.47496 (14)	0.34122 (8)	0.56039 (12)	0.0555 (7)	
C22	0.3423 (3)	0.41754 (16)	0.8371 (3)	0.0691 (9)	
H22	0.3702	0.4479	0.8581	0.083*	
C23	0.24100 (14)	0.36003 (8)	0.80981 (14)	0.0602 (8)	
C24	0.16767 (11)	0.32924 (11)	0.80189 (16)	0.0787 (12)	
H24	0.1166	0.3415	0.8255	0.094*	
C25	0.17062 (13)	0.28005 (11)	0.75867 (18)	0.0788 (10)	
H25	0.1216	0.2595	0.7534	0.095*	
C26	0.24690 (16)	0.26166 (8)	0.72338 (16)	0.0724 (10)	
H26	0.2489	0.2288	0.6945	0.087*	
C27	0.32023 (12)	0.29245 (8)	0.73130 (14)	0.0616 (8)	
H27	0.3713	0.2801	0.7077	0.074*	
C28	0.31728 (11)	0.34163 (8)	0.77451 (14)	0.0550 (7)	

### Atomic displacement parameters (Å^2^)

**Table:**

	*U*^11^	*U*^22^	*U*^33^	*U*^12^	*U*^13^	*U*^23^
Cu1	0.0294 (2)	0.0434 (2)	0.0389 (2)	0.000	−0.0016 (2)	0.000
Cu2	0.0546 (3)	0.0549 (3)	0.0566 (3)	0.000	0.0062 (4)	0.000
O1	0.068 (4)	0.073 (4)	0.044 (3)	0.014 (3)	−0.013 (3)	−0.021 (3)
O2	0.115 (8)	0.055 (2)	0.134 (7)	0.020 (3)	−0.018 (6)	0.000 (4)
O3	0.086 (5)	0.091 (5)	0.044 (3)	0.008 (4)	−0.009 (4)	−0.006 (3)
O4	0.129 (6)	0.060 (2)	0.133 (5)	−0.009 (3)	−0.036 (6)	0.003 (5)
O5	0.118 (6)	0.138 (6)	0.211 (9)	0.052 (5)	0.003 (7)	−0.033 (7)
O6	0.061 (4)	0.142 (6)	0.147 (6)	−0.025 (4)	0.007 (4)	0.027 (5)
O7	0.089 (6)	0.090 (6)	0.089 (6)	−0.006 (6)	0.003 (5)	0.001 (5)
O8	0.057 (3)	0.101 (4)	0.174 (8)	−0.019 (4)	0.015 (4)	−0.031 (6)
O9	0.063 (4)	0.095 (5)	0.090 (5)	−0.007 (3)	0.019 (4)	0.011 (4)
O10	0.046 (4)	0.073 (6)	0.050 (7)	0.006 (4)	0.003 (4)	−0.012 (6)
O11	0.0424 (17)	0.071 (6)	0.078 (9)	0.005 (4)	−0.006 (3)	0.018 (6)
O12	0.061 (5)	0.073 (6)	0.043 (6)	−0.007 (4)	0.010 (4)	−0.006 (4)
N1	0.0368 (10)	0.0522 (12)	0.0380 (10)	0.0020 (9)	−0.0027 (9)	−0.0038 (9)
N2	0.0533 (14)	0.0601 (14)	0.0394 (12)	−0.0063 (12)	0.0041 (11)	−0.0073 (11)
N3	0.0311 (10)	0.0538 (12)	0.0459 (11)	0.0016 (10)	−0.0018 (9)	0.0093 (10)
N4	0.0348 (13)	0.0754 (18)	0.113 (3)	−0.0051 (12)	−0.0058 (15)	0.0349 (19)
N5	0.0558 (15)	0.0634 (15)	0.0617 (16)	0.0083 (13)	0.0050 (14)	0.0001 (13)
N6	0.0720 (19)	0.078 (2)	0.0736 (19)	0.0117 (17)	−0.0163 (17)	0.0069 (16)
N7	0.0567 (15)	0.0606 (15)	0.0621 (16)	0.0058 (13)	0.0081 (13)	−0.0081 (13)
N8	0.075 (2)	0.0740 (19)	0.0630 (16)	0.0228 (16)	0.0177 (16)	−0.0025 (15)
N9	0.054 (2)	0.0477 (17)	0.0371 (15)	−0.017 (5)	−0.0012 (19)	−0.002 (8)
N10	0.052 (2)	0.058 (2)	0.069 (7)	0.016 (4)	0.019 (4)	−0.007 (3)
N11	0.052 (3)	0.061 (4)	0.084 (9)	0.003 (4)	0.006 (3)	0.016 (7)
N12	0.048 (2)	0.0502 (19)	0.047 (6)	−0.009 (6)	−0.016 (6)	0.009 (4)
C1	0.0430 (14)	0.0548 (13)	0.0455 (13)	−0.0012 (14)	0.0056 (14)	−0.0086 (11)
C2	0.0499 (15)	0.0543 (15)	0.0441 (14)	−0.0120 (13)	−0.0027 (13)	0.0022 (12)
C3	0.077 (2)	0.075 (2)	0.0459 (16)	−0.0086 (19)	−0.0147 (17)	0.0073 (16)
C4	0.070 (2)	0.084 (2)	0.062 (2)	0.0121 (19)	−0.0175 (18)	0.0206 (19)
C5	0.066 (2)	0.073 (2)	0.066 (2)	0.0192 (19)	0.0028 (17)	0.0113 (17)
C6	0.0521 (17)	0.0542 (16)	0.0483 (15)	0.0105 (14)	0.0007 (13)	0.0039 (13)
C7	0.0389 (13)	0.0467 (13)	0.0428 (13)	−0.0043 (11)	−0.0016 (12)	0.0023 (11)
C8	0.0377 (13)	0.0582 (15)	0.0730 (18)	−0.0052 (11)	−0.0081 (16)	0.0109 (19)
C9	0.0403 (14)	0.0627 (17)	0.0498 (15)	0.0143 (13)	0.0072 (13)	0.0207 (14)
C10	0.074 (2)	0.075 (2)	0.0479 (16)	0.0285 (19)	−0.0030 (17)	0.0040 (16)
C11	0.110 (4)	0.098 (3)	0.064 (2)	0.060 (3)	0.017 (2)	0.008 (2)
C12	0.107 (4)	0.108 (4)	0.081 (3)	0.052 (3)	0.040 (3)	0.023 (3)
C13	0.055 (2)	0.127 (4)	0.145 (5)	0.043 (3)	0.049 (3)	0.080 (4)
C14	0.0476 (17)	0.078 (2)	0.079 (2)	0.0113 (16)	0.0109 (17)	0.035 (2)
C15	0.064 (2)	0.066 (2)	0.085 (3)	0.0150 (18)	−0.003 (2)	−0.005 (2)
C16	0.0464 (16)	0.073 (2)	0.072 (2)	−0.0007 (15)	−0.0037 (16)	0.0002 (18)
C17	0.065 (2)	0.117 (3)	0.063 (2)	−0.009 (2)	−0.0083 (19)	−0.014 (2)
C18	0.062 (2)	0.098 (3)	0.091 (3)	−0.002 (2)	0.002 (2)	−0.033 (2)
C19	0.060 (2)	0.069 (2)	0.100 (3)	−0.0017 (17)	−0.002 (2)	−0.019 (2)
C20	0.057 (2)	0.0583 (17)	0.076 (2)	0.0025 (15)	0.0054 (16)	0.0019 (15)
C21	0.0445 (16)	0.0617 (17)	0.0604 (17)	−0.0052 (13)	0.0073 (13)	−0.0039 (15)
C22	0.078 (2)	0.0638 (19)	0.066 (2)	0.0076 (19)	0.0103 (19)	−0.0075 (17)
C23	0.0575 (19)	0.078 (2)	0.0449 (15)	0.0174 (17)	0.0030 (14)	0.0051 (15)
C24	0.0525 (19)	0.124 (4)	0.059 (2)	0.008 (2)	0.0070 (16)	0.018 (2)
C25	0.074 (2)	0.101 (3)	0.061 (2)	−0.016 (2)	−0.004 (2)	0.004 (2)
C26	0.083 (2)	0.079 (2)	0.0543 (18)	−0.012 (2)	0.0025 (18)	0.0043 (16)
C27	0.065 (2)	0.0661 (19)	0.0533 (19)	0.0039 (16)	0.0013 (15)	−0.0012 (14)
C28	0.0525 (17)	0.0644 (18)	0.0481 (15)	0.0077 (15)	0.0012 (13)	0.0038 (13)

### Geometric parameters (Å, °)

**Table:**

Cu1—N1^i^	1.998 (2)	C2—C3	1.3900
Cu1—N1	1.998 (2)	C2—C7	1.3900
Cu1—N3	2.008 (2)	C3—C4	1.3900
Cu1—N3^i^	2.008 (2)	C3—H3	0.9300
Cu1—O1	2.590 (7)	C4—C5	1.3900
Cu2—N5	1.989 (3)	C4—H4A	0.9300
Cu2—N5^ii^	1.989 (3)	C5—C6	1.3900
Cu2—N7^ii^	1.993 (3)	C5—H5	0.9300
Cu2—N7	1.993 (3)	C6—C7	1.3900
Cu2—O4	2.468 (5)	C6—H6A	0.9300
O1—N9	1.240 (7)	C8—H8A	0.9300
O2—N9	1.218 (5)	C9—C10	1.3900
O3—N9	1.236 (9)	C9—C14	1.3900
O4—N10	1.205 (6)	C10—C11	1.3900
O5—N10	1.234 (7)	C10—H10	0.9300
O6—N10	1.237 (8)	C11—C12	1.3900
O7—N11	1.244 (8)	C11—H11	0.9300
O8—N11	1.231 (7)	C12—C13	1.3900
O9—N11	1.246 (8)	C12—H12	0.9300
O10—N12	1.250 (8)	C13—C14	1.3900
O11—N12	1.240 (6)	C13—H13	0.9300
O12—N12	1.239 (8)	C15—H15	0.9300
N1—C1	1.334 (4)	C16—C17	1.3900
N1—C7	1.382 (3)	C16—C21	1.3900
N2—C1	1.330 (4)	C17—C18	1.3900
N2—C2	1.348 (3)	C17—H17	0.9300
N2—H2	0.8800	C18—C19	1.3900
N3—C8	1.325 (4)	C18—H18	0.9300
N3—C9	1.388 (3)	C19—C20	1.3900
N4—C8	1.346 (4)	C19—H19	0.9300
N4—C14	1.368 (4)	C20—C21	1.3900
N4—H4	0.8800	C20—H20	0.9300
N5—C15	1.301 (5)	C22—H22	0.9300
N5—C21	1.370 (3)	C23—C24	1.3900
N6—C15	1.338 (6)	C23—C28	1.3900
N6—C16	1.354 (4)	C24—C25	1.3900
N6—H6	0.8800	C24—H24	0.9300
N7—C22	1.320 (5)	C25—C26	1.3900
N7—C28	1.379 (3)	C25—H25	0.9300
N8—C22	1.337 (5)	C26—C27	1.3900
N8—C23	1.365 (4)	C26—H26	0.9300
N8—H8	0.8800	C27—C28	1.3900
C1—H1	0.9300	C27—H27	0.9300
			
N1^i^—Cu1—N1	164.10 (14)	C4—C5—H5	120.0
N1^i^—Cu1—N3	91.08 (10)	C6—C5—H5	120.0
N1—Cu1—N3	89.06 (10)	C7—C6—C5	120.0
N1^i^—Cu1—N3^i^	89.06 (10)	C7—C6—H6A	120.0
N1—Cu1—N3^i^	91.08 (10)	C5—C6—H6A	120.0
N3—Cu1—N3^i^	179.02 (14)	N1—C7—C6	131.44 (15)
N1^i^—Cu1—O1	77.06 (17)	N1—C7—C2	108.53 (15)
N1—Cu1—O1	118.83 (17)	C6—C7—C2	120.0
N3—Cu1—O1	88.23 (19)	N3—C8—N4	110.7 (3)
N3^i^—Cu1—O1	90.9 (2)	N3—C8—H8A	124.6
N5—Cu2—N5^ii^	176.00 (17)	N4—C8—H8A	124.6
N5—Cu2—N7^ii^	89.60 (13)	N3—C9—C10	131.12 (17)
N5^ii^—Cu2—N7^ii^	90.27 (13)	N3—C9—C14	108.88 (17)
N5—Cu2—N7	90.27 (13)	C10—C9—C14	120.0
N5^ii^—Cu2—N7	89.60 (13)	C9—C10—C11	120.0
N7^ii^—Cu2—N7	176.34 (17)	C9—C10—H10	120.0
N5—Cu2—O4	87.0 (3)	C11—C10—H10	120.0
N5^ii^—Cu2—O4	97.0 (3)	C10—C11—C12	120.0
N7^ii^—Cu2—O4	100.5 (2)	C10—C11—H11	120.0
N7—Cu2—O4	83.2 (2)	C12—C11—H11	120.0
N9—O1—Cu1	111.0 (5)	C13—C12—C11	120.0
N10—O4—Cu2	146.3 (6)	C13—C12—H12	120.0
C1—N1—C7	105.0 (2)	C11—C12—H12	120.0
C1—N1—Cu1	123.3 (2)	C12—C13—C14	120.0
C7—N1—Cu1	131.62 (17)	C12—C13—H13	120.0
C1—N2—C2	108.1 (2)	C14—C13—H13	120.0
C1—N2—H2	125.9	N4—C14—C13	134.80 (19)
C2—N2—H2	125.9	N4—C14—C9	105.18 (19)
C8—N3—C9	106.2 (2)	C13—C14—C9	120.0
C8—N3—Cu1	122.2 (2)	N5—C15—N6	112.4 (4)
C9—N3—Cu1	131.17 (17)	N5—C15—H15	123.8
C8—N4—C14	109.0 (3)	N6—C15—H15	123.8
C8—N4—H4	125.5	N6—C16—C17	134.4 (2)
C14—N4—H4	125.5	N6—C16—C21	105.6 (2)
C15—N5—C21	105.8 (3)	C17—C16—C21	120.0
C15—N5—Cu2	123.6 (3)	C16—C17—C18	120.0
C21—N5—Cu2	130.3 (2)	C16—C17—H17	120.0
C15—N6—C16	107.5 (3)	C18—C17—H17	120.0
C15—N6—H6	126.3	C19—C18—C17	120.0
C16—N6—H6	126.3	C19—C18—H18	120.0
C22—N7—C28	105.7 (3)	C17—C18—H18	120.0
C22—N7—Cu2	124.5 (3)	C20—C19—C18	120.0
C28—N7—Cu2	129.45 (19)	C20—C19—H19	120.0
C22—N8—C23	107.8 (3)	C18—C19—H19	120.0
C22—N8—H8	126.1	C19—C20—C21	120.0
C23—N8—H8	126.1	C19—C20—H20	120.0
O2—N9—O3	121.8 (8)	C21—C20—H20	120.0
O2—N9—O1	120.7 (9)	N5—C21—C20	131.35 (19)
O3—N9—O1	117.5 (5)	N5—C21—C16	108.65 (19)
O4—N10—O5	118.7 (9)	C20—C21—C16	120.0
O4—N10—O6	123.0 (7)	N7—C22—N8	112.0 (4)
O5—N10—O6	118.2 (7)	N7—C22—H22	124.0
O8—N11—O7	120.0 (7)	N8—C22—H22	124.0
O8—N11—O9	119.3 (7)	N8—C23—C24	134.2 (2)
O7—N11—O9	120.4 (7)	N8—C23—C28	105.8 (2)
O11—N12—O12	120.5 (10)	C24—C23—C28	120.0
O11—N12—O10	119.7 (10)	C23—C24—C25	120.0
O12—N12—O10	118.6 (6)	C23—C24—H24	120.0
N2—C1—N1	112.0 (3)	C25—C24—H24	120.0
N2—C1—H1	124.0	C26—C25—C24	120.0
N1—C1—H1	124.0	C26—C25—H25	120.0
N2—C2—C3	133.68 (17)	C24—C25—H25	120.0
N2—C2—C7	106.31 (17)	C27—C26—C25	120.0
C3—C2—C7	120.0	C27—C26—H26	120.0
C2—C3—C4	120.0	C25—C26—H26	120.0
C2—C3—H3	120.0	C26—C27—C28	120.0
C4—C3—H3	120.0	C26—C27—H27	120.0
C3—C4—C5	120.0	C28—C27—H27	120.0
C3—C4—H4A	120.0	N7—C28—C27	131.33 (18)
C5—C4—H4A	120.0	N7—C28—C23	108.66 (18)
C4—C5—C6	120.0	C27—C28—C23	120.0
			
N1^i^—Cu1—O1—N9	177.7 (5)	Cu1—N3—C8—N4	175.3 (2)
N1—Cu1—O1—N9	−2.8 (5)	C14—N4—C8—N3	−0.6 (4)
N3—Cu1—O1—N9	−90.8 (4)	C8—N3—C9—C10	178.5 (2)
N3^i^—Cu1—O1—N9	88.9 (4)	Cu1—N3—C9—C10	5.7 (3)
N5—Cu2—O4—N10	−139.8 (18)	C8—N3—C9—C14	−2.2 (3)
N5^ii^—Cu2—O4—N10	40.8 (18)	Cu1—N3—C9—C14	−174.97 (16)
N7^ii^—Cu2—O4—N10	−50.8 (18)	N3—C9—C10—C11	179.3 (2)
N7—Cu2—O4—N10	129.6 (18)	C14—C9—C10—C11	0.0
N1^i^—Cu1—N1—C1	−136.0 (2)	C9—C10—C11—C12	0.0
N3—Cu1—N1—C1	133.4 (2)	C10—C11—C12—C13	0.0
N3^i^—Cu1—N1—C1	−45.7 (2)	C11—C12—C13—C14	0.0
O1—Cu1—N1—C1	45.9 (3)	C8—N4—C14—C13	−179.2 (2)
N1^i^—Cu1—N1—C7	49.0 (2)	C8—N4—C14—C9	−0.8 (3)
N3—Cu1—N1—C7	−41.7 (2)	C12—C13—C14—N4	178.3 (3)
N3^i^—Cu1—N1—C7	139.3 (2)	C12—C13—C14—C9	0.0
O1—Cu1—N1—C7	−129.1 (3)	N3—C9—C14—N4	1.8 (2)
N1^i^—Cu1—N3—C8	146.8 (2)	C10—C9—C14—N4	−178.7 (2)
N1—Cu1—N3—C8	−49.1 (2)	N3—C9—C14—C13	−179.4 (2)
O1—Cu1—N3—C8	69.8 (3)	C10—C9—C14—C13	0.0
N1^i^—Cu1—N3—C9	−41.4 (2)	C21—N5—C15—N6	−1.3 (5)
N1—Cu1—N3—C9	122.7 (2)	Cu2—N5—C15—N6	173.2 (3)
O1—Cu1—N3—C9	−118.4 (3)	C16—N6—C15—N5	1.2 (5)
N7^ii^—Cu2—N5—C15	−115.3 (3)	C15—N6—C16—C17	178.9 (3)
N7—Cu2—N5—C15	68.3 (3)	C15—N6—C16—C21	−0.5 (4)
O4—Cu2—N5—C15	−14.8 (4)	N6—C16—C17—C18	−179.4 (3)
N7^ii^—Cu2—N5—C21	57.7 (3)	C21—C16—C17—C18	0.0
N7—Cu2—N5—C21	−118.6 (3)	C16—C17—C18—C19	0.0
O4—Cu2—N5—C21	158.2 (4)	C17—C18—C19—C20	0.0
N5—Cu2—N7—C22	−116.5 (3)	C18—C19—C20—C21	0.0
N5^ii^—Cu2—N7—C22	67.5 (3)	C15—N5—C21—C20	−178.8 (3)
O4—Cu2—N7—C22	−29.6 (4)	Cu2—N5—C21—C20	7.2 (4)
N5—Cu2—N7—C28	55.6 (3)	C15—N5—C21—C16	0.9 (3)
N5^ii^—Cu2—N7—C28	−120.4 (3)	Cu2—N5—C21—C16	−173.1 (2)
O4—Cu2—N7—C28	142.5 (4)	C19—C20—C21—N5	179.8 (3)
Cu1—O1—N9—O2	−165.0 (7)	C19—C20—C21—C16	0.0
Cu1—O1—N9—O3	15.0 (7)	N6—C16—C21—N5	−0.3 (3)
Cu2—O4—N10—O5	−159.8 (14)	C17—C16—C21—N5	−179.8 (2)
Cu2—O4—N10—O6	17 (2)	N6—C16—C21—C20	179.6 (2)
C2—N2—C1—N1	2.0 (3)	C17—C16—C21—C20	0.0
C7—N1—C1—N2	−0.2 (3)	C28—N7—C22—N8	−0.5 (4)
Cu1—N1—C1—N2	−176.4 (2)	Cu2—N7—C22—N8	173.2 (3)
C1—N2—C2—C3	178.3 (2)	C23—N8—C22—N7	0.3 (4)
C1—N2—C2—C7	−2.8 (3)	C22—N8—C23—C24	178.5 (2)
N2—C2—C3—C4	178.8 (3)	C22—N8—C23—C28	0.0 (3)
C7—C2—C3—C4	0.0	N8—C23—C24—C25	−178.3 (3)
C2—C3—C4—C5	0.0	C28—C23—C24—C25	0.0
C3—C4—C5—C6	0.0	C23—C24—C25—C26	0.0
C4—C5—C6—C7	0.0	C24—C25—C26—C27	0.0
C1—N1—C7—C6	−179.5 (2)	C25—C26—C27—C28	0.0
Cu1—N1—C7—C6	−3.8 (3)	C22—N7—C28—C27	−178.4 (2)
C1—N1—C7—C2	−1.6 (2)	Cu2—N7—C28—C27	8.3 (4)
Cu1—N1—C7—C2	174.14 (17)	C22—N7—C28—C23	0.5 (3)
C5—C6—C7—N1	177.7 (2)	Cu2—N7—C28—C23	−172.7 (2)
C5—C6—C7—C2	0.0	C26—C27—C28—N7	178.8 (3)
N2—C2—C7—N1	2.7 (2)	C26—C27—C28—C23	0.0
C3—C2—C7—N1	−178.2 (2)	N8—C23—C28—N7	−0.3 (2)
N2—C2—C7—C6	−179.1 (2)	C24—C23—C28—N7	−179.1 (2)
C3—C2—C7—C6	0.0	N8—C23—C28—C27	178.7 (2)
C9—N3—C8—N4	1.7 (4)	C24—C23—C28—C27	0.0

Symmetry codes: (i) −*x*, *y*, −*z*+1/2; (ii) −*x*+1, *y*, −*z*+3/2.

### Hydrogen-bond geometry (Å, °)

**Table:**

*D*—H···*A*	*D*—H	H···*A*	*D*···*A*	*D*—H···*A*
N2—H2···O1^iii^	0.88	2.21	3.069 (9)	167
N2—H2···O3^iv^	0.88	1.89	2.742 (9)	164
N4—H4···O5^iv^	0.88	2.24	2.964 (9)	139
N4—H4···O6^v^	0.88	2.10	2.955 (7)	163
N6—H6···O7	0.88	2.23	2.998 (14)	146
N8—H8···O10	0.88	1.91	2.79 (2)	173
N8—H8···O12^vi^	0.88	1.87	2.75 (3)	175

Symmetry codes: (iii) −*x*, −*y*+1, *z*+1/2; (iv) *x*, −*y*+1, −*z*+1; (v) −*x*+1, −*y*+1, *z*−1/2; (vi) *x*, −*y*+1, −*z*+2.

## References

[bb1] Agilent (2010). *CrysAlis PRO* Agilent Technologies, Yarnton, Oxfordshire, England.

[bb2] Barbour, L. J. (2001). *J. Supramol. Chem.* **1**, 189–191.

[bb3] Dobrzyńska, D., Janczak, J., Wojciechowska, A. & Helios, K. (2010). *J. Mol. Struct.* **973**, 62–68.

[bb4] Flack, H. D. (1983). *Acta Cryst.* A**39**, 876–881.

[bb5] McFadden, D. L., McPhail, A. T., Garner, C. D. & Mabbs, F. E. (1975). *J. Chem. Soc. Dalton Trans.* pp. 263–268.

[bb6] McFadden, D. L., McPhail, A. T., Gross, P. M., Garner, C. D. & Mabbs, F. E. (1976). *J. Chem. Soc. Dalton Trans.* pp. 47–52.

[bb7] Sheldrick, G. M. (2008). *Acta Cryst.* A**64**, 112–122. 10.1107/S0108767307043930 18156677

[bb8] Sieroń, L. (2007). *Acta Cryst.* E**63**, m579–m580.

[bb9] Westrip, S. P. (2010). *J. Appl. Cryst.* **43**, 920–925.

